# Intrinsic and extrinsic risk factors in tumor-related epilepsy

**DOI:** 10.1007/s10143-025-04020-z

**Published:** 2025-12-26

**Authors:** Omar Rafi, Alessandro Carretta, Luca Zanuttini, Victor Staartjes, Flavio Vasella, Stefanos Voglis, Michael Weller, Niklaus Krayenbühl, Luca Regli, Carlo Serra, Kevin Akeret

**Affiliations:** 1 Department of Neurosurgery, Clinical Neuroscience Center, University Hospital Zurich, University of Zurich, Zurich, Switzerland; 2 Department of Biomedical and Neuromotor Sciences (DIBINEM), University of Bologna, Bologna, Italy; 3 Department of Neurology, Clinical Neuroscience Center, University Hospital Zurich, University of Zurich, Zurich, Switzerland; 4 Division of Pediatric Neurosurgery, University Children’s Hospital, Zurich, Switzerland

**Keywords:** Anatomy, Glioma, Metastases, Seizure, Topography

## Abstract

**Purpose:**

This study consolidated evidence on the association of tumor-intrinsic and investigated the underexplored association of tumor-extrinsic factors with tumor-related epilepsy, aiming to contribute to the overall risk assessment for clinical management in tumor-related epilepsy.

**Material & methods:**

Over a period of one year (2020), we prospectively collected imaging and clinical data of 373 patients with histopathologically confirmed brain tumors. Assessed tumor-intrinsic factors included histopathology, diameter and anatomical location, with the central lobe comprising the precentral, postcentral and subcentral gyrus and paracentral lobule. Tumor-extrinsic factors comprised sex, age, BMI, smoking, alcohol abuse, and Na^+^/K^+^ imbalances. We applied univariable and multivariable binary logistic regression to characterize the associations between tumor-intrinsic and tumor-extrinsic factors with tumor-related epilepsy.

**Results:**

Preoperative seizures occurred in 29.5% (*n* = 110) of patients, with cortical locations — particularly the central lobe (91.3%, *n* = 21) — posing the highest seizure incidence among all factors. In univariable analysis, compared to WHO grade 3/4 gliomas, WHO grade 1/2 neuroepithelial tumors exhibited moderately higher, whereas pituitary adenomas/ craniopharyngiomas and schwannomas showed lower incidences of preoperative seizures. In multivariable analysis, the central lobe (OR 58.15), other cortical locations (OR 3.47–3.76), male sex (OR 1.77), low BMI (OR 4.61) and smoking (OR 1.013 per pack-year), revealed significant associations (p = < 0.05).

**Conclusion:**

Cortical location, especially in the central lobe, male sex, low BMI and smoking, are independently associated with tumor-related epilepsy. Our findings support the importance of considering both tumor-intrinsic and tumor-extrinsic factors to develop a holistic seizure-risk assessment and highlight the need for larger, prospective studies to refine clinical management and potentially pharmacological seizure prophylaxis.

**Supplementary Information:**

The online version contains supplementary material available at 10.1007/s10143-025-04020-z.

## Introduction

Tumor-related epilepsy (TRE) represents a frequent and debilitating neurological complication, further exacerbating the clinical burden and quality-of-life impairment in patients with brain tumors [1]. Nevertheless, due to well-known side effects of antiseizure medication and an incomplete understanding of TRE’s pathophysiology and seizure risk stratification, the prophylactic use of antiseizure medication is generally not recommended [2–5]. Further insights into all seizure risk factors, including less-studied tumor-extrinsic factors (TEFs), are needed. These insights could enhance clinical management and reveal potential benefits through lifestyle changes [2, 3, 6, 7].

The most prominent known tumor-intrinsic factor (TIF) is anatomical location, with particularly the temporal and frontal lobes associated with higher risks [1, 7–14]. However, the anatomical distinction of a central lobe [15, 16], as opposed to the traditional frontal-parietal lobe concept, appears especially relevant in the context of TRE, given its strong observed association with seizure incidence [11, 12]. Other TIFs such as histopathology have also exhibit associations with higher incidences of 60–100% in patients with WHO grade 1/2 neuroepithelial tumors and rather low incidences in patients with WHO grade 3/4 gliomas between 30 and 50% [8, 11, 12, 17]. In contrast, associaions of seizure risk with tumor size remain controversial [18–20].

Certain TEFs like sex, age, lifestyle, and pathological laboratory levels may also be related to TRE. However, current data on this is limited, with only some indications that younger age and male sex might be associated with higher seizure risks [20–22]. Further investigation into these factors is particularly important, as they could serve for initial risk stratification in clinical practice and are potentially modifiable.

The objective of this study was to deepen our insights into the relevance of competitive or synergistic TIFs and TEFs. Therefore, we aimed to screen for factors with the strongest associations, emphasize anatomical predispositions linked to TRE, identify clinically relevant and early detectable TEFs, and thus contribute to a more comprehensive framework for risk stratification

## Material & Methods

### Study population

During 2020, we conducted a weekly standardized approach to collect epidemiologic, clinical, imaging, and histopathological data for all patients undergoing brain tumor surgery. The final inclusion criteria were: (I) histopathological diagnosis of a primary or secondary brain tumor after resection or biopsy; (II) no use of prophylactic antiseizure medication; (III) availability of a standardized preoperative MRI (for technical details, see *Supplementary Methods*). Patients with inconclusive histopathological results, secondary intracranial pathology such as vascular or infectious diseases, or a history of previous structural lesions or cranial surgery were excluded.

### Data collection

Ethics board approval was obtained prior to data gathering from the Cantonal Ethics Committee of Zurich (KEK-ZH-Nr. 01120). Informed general consent for scientific use of all medical data was obtained from all patients upon admission to the hospital. Clinical trial number: Not applicable.

The definitive histopathological diagnosis was made after biopsy or surgery by independent analysis from our Neuropathology department and categorized according to the *2021 WHO Classification of Tumors of the Central Nervous System* [23]. Two neurosurgeons (CS, AC) independently collected and analyzed topographical data using standard morphological MR sequences (T1, T1 with contrast, T2, FLAIR), being blinded to the histopathological and tumor-extrinsic data. The standard approach was to describe infiltrated, but not displaced or edematous structures due to methodological heterogeneity. Imaging data were analyzed for following tumor features: (I) diameter; (II) intraparenchymal uni- vs. multifocality (included larger tumors encompassing multiple regions), and if unifocal; (III) exact lobar location; or (IV) extraparenchymal location. We applied the concept of a central lobe on a lobar level, composed of the precentral, postcentral and subcentral gyrus and paracentral lobule [15, 16], excluding these areas in the frontal and parietal lobes.

TRE was defined as seizures occurring preoperatively without prior antiseizure medication. The presence or absence of TRE was collected as a binary categorical variable (yes vs. no). The collected TEFs included demographic characteristics (sex and age), lifestyle factors (BMI, smoking behavior [in pack-years] and alcohol abuse) and the clinical most relevant electrolyte disturbances, particularly pathological Na^+^/K^+^ levels. While age and pack-years were recorded as continuous data, sex (male vs. female), BMI categories (underweight [BMI < 18.5], normal weight [BMI 18.5–24.9], overweight [BMI 25–29.9], obese [BMI ≥ 30]), alcohol abuse (yes vs. no) or pathological Na^+^/K^+^ levels (yes vs. no) were collected as categorical variables. Normal Na^+^/K^+^ levels were defined as 135–145 mmol/L and 3.5–5.0 mmol/L. The most recent result before a seizure was used; if unavailable, the pre-surgery blood result was selected.

### Statistical Analysis

The primary objective of our study was to describe the association of the incidence of preoperative TRE (dependent variable) with independent variables including TIFs (histopathology, tumor diameter, anatomical location) and TEFs (sex, age, BMI, smoking, alcohol abuse, pathological Na^+^/K^+^ levels). The following statistical approach was applied to assess the association of TIFs and TEFs with TRE: (I) crosstabs provided a descriptive overview of the quantitative distribution of subgroups within each variable. If counts were < 10, expressions were combined or merged with an existing group; (II) seizure risk was calculated, and univariable binary logistic regression assessed the isolated effect size (odds ratio [OR], 95% confidence intervals [CI], p value); (III) for variables with multiple expressions, the most frequent was generally chosen as the reference level, unless subgroup sizes were similar, in which case a content-related reference was selected; (IV) only statistically significant TIFs and TEFs (*p* < 0.05) were included in the multivariable binary logistic regression model to assess the real effect size and interactions, but also to prevent overfitting and to improve model stability by reducing noise and unnecessary predictors. Significant factors in this analysis were interpreted having independent associations with TRE; (V) to prepare for combining relevant TEFs in a heatmap, interaction effects were tested using hierarchical logistic regression. A model with main effects was compared to one including all two- and three-way interactions via chi-square deviance tests. This methodological prerequisite showed no significant improvement (*p* > 0.05), indicating no significant interactions. Continuous variables were displayed as means ± standard deviations and categorical data as absolute numbers and percentages with CIs. Analyses were conducted and figures were generated using R 4.4.1 and RStudio 2024.04.2 + 764.

## Results

All descriptive data and the results from univariable analysis are displayed in Table 1.. Of 373 patients 194 patients (52%) were female, the mean age was 55 years, 176 patients were of normal weight (47.2%) and 126 patients (33.8%) were overweight. Patients had an average of 11.5 smoking pack-years (SD 20.9), while 5.7% had a history of alcohol abuse, and 19.9% presented with pathological Na^+^/K^+^ levels either during hospitalization or near a seizure event.

**Table 1.** TEFs and TIFs of patients containing primary or secondary brain tumor

Risk factor	Subgroup prevalence	Cat.: N/TS, Cont.: Mean (SD)	Seizure incidence (CI 95%)	Odds ratio (CI 95%)	P value
	% (= TS/TP)				
***Tumor-extrinsic***					
**Sex**					
Female (ref.)	52%	46/194	23.7 % (23.1 - 24.3)	1 (ref.)	
Male	48%	64/179	35.8 % (35.1 - 36.5)	1.79 (1.14 - 2.82)	**0.0113**
Age (cont.)		55.7 (15.7)		0.998 (0.984 - 1.012)	0.8189
		55.3 (15.3)			
**BMI groups**					
≥ 30: Obese (ref.)	13.40%	7/50	14 % (13 - 15)	1 (ref.)	
25 - 29.9: Overweight	33.80%	32/126	25.4 % (24.6 - 26.2)	2.09 (0.9 - 5.5)	0.1058
18.5 - 24.9: Normal	47.20%	62/176	35.2 % (34.5 - 35.9)	3.34 (1.5 - 8.52)	**0.0058**
< 18.5: Underweight	5.60%	9/21	42.9 % (40.7 - 45)	4.61 (1.44 - 15.54)	**0.011**
**Alcohol abuse**					
No (ref.)	94.30%	101/352	28.7 % (28.2 - 29.2)	1 (ref.)	
Yes	5.60%	9/21	42.9 % (40.7 - 45)	1.86 (0.74 - 4.54)	0.1725
Smoking pack-years (cont.)		9.4 (18.5)		1.015 (1.005 - 1.026)	**0.003**
		16.7 (25)			
Pathological Na^+^/K^+^blood value					
No (ref.)	80.10%	81/299	27.1 % (26.6 - 27.6)	1 (ref.)	
Yes	19.90%	29/74	39.2 % (38.1 - 40.3)	1.73 (1.01 - 2.94)	**0.0424**
*Tumor-intrinsic*					
**Pathology**					
WHO grade 3/4 glioma (ref.)	26.30%	32/98	32.7 % (31.7 - 33.6)	1 (ref.)	
WHO grade 1/2 neuroepith. tumor	4.30%	7/16	43.6 % (41.3 - 46.2)	1.61 (0.53 - 4.70)	0.3885
Pit. adenoma/craniopharyngioma	8.60%	2/32	6.3 % (5.4 - 7.1)	0.14 (0.02 - 0.50)	**0.0092**
Primary CNS Lymphoma	3.50%	5/13	38.5 % (35.8 - 41.1)	1.29 (0.36 - 4.18)	0.6769
Meningioma	21.70%	19/81	23.5 % (22.5 - 24.4)	0.63 (0.32 - 1.22)	0.1764
Metastasis	21.50%	32/80	40.0 % (38.9 - 41.1)	1.38 (0.74 - 2.55)	0.3102
Schwannoma	3.60%	1/14	7.1 % (5.8 - 8.5)	0.16 (0.01 - 0.85)	0.0824
Tumor otherwise	10.50%	12/39	30.8 % (29.3 - 32.2)	0.92 (0.40 - 2.01)	0.8313
Diameter mm (cont.)		36.2 (17.3)		1.00 (0.987 - 1.014)	0.9425
		36.3 (16.9)			
**Location**					
Other (ref.)	13.40%	7/50	14 % (4.4 - 23.6)	1 (ref.)	
Frontal lobe	9.70%	13/36	36.1 % (20.4 - 51.8)	3.47 (1.25 - 10.40)	**0.02**
Parietal lobe	6.40%	9/24	37.5 % (18.1 - 56.9)	3.69 (1.18 - 12.06)	**0.0261**
Temporal lobe	12.90%	17/48	35.4 % (21.9 - 48.9)	3.37 (1.29 - 9.63)	**0.0166**
Occipital lobe	3.80%	4/14	28.6 % (4.9 - 52.2)	2.46 (0.56 - 9.92)	0.2108
Central lobe	6.10%	21/23	91.3 % (79.8 - 100)	64.5 (14.88 - 466.3)	**< 0.0001**
Cerebellum	4.80%	1/18	5.6 % (0.0 - 16.1)	0.36 (0.02 - 2.25)	0.3577
Multifocal (intraparenchymal)	11%	17/41	41.5 % (26.4 - 56.5)	4.35 (1.64 - 12.66)	**0.0044**
Convexity (osseus/meningeal)	10.50%	12/39	30.8 % (16.3 - 45.3)	2.73 (0.98 - 8.16)	0.0606
Intracranial dural folds	4.30%	5/16	31.3 % (8.5 - 54)	2.79 (0.71 - 10.56)	0.1288
Anterior cranial fossa	4%	1/15	6.7 % (0.0 - 19.3)	0.44 (0.02 - 2.78)	0.459
Middle cranial fossa	5.60%	2/12	9.5 % (0.0 - 22.1)	0.65 (0.09 - 2.98)	0.6071
Posterior cranial fossa	7.50%	1/18	3.6 % (0.0 - 10.4)	0.23 (0.01 - 1.38)	0.1769

Total patients (TP) = 373. Total subgroup quantity (TS)

Categorial variables: Subgroup prevalence, affected individuals (N) and seizure incidence (%) including confidence interval (CI)

Continuous variables: Means of non-affected (above) vs. affected (below) individuals including standard deviation (SD)

Both: Odds ratios (OR) including CI and p value from univariable binary logistic regression

Most patients suffered from WHO grade 3/4 gliomas (*n* = 98, 26.3%), meningioma (*n* = 81, 21.7%), or metastasis (*n* = 80, 21.5%), while fewer patients had WHO grade 1/2 neuroepithelial tumors (*n* = 16, 4.3%), schwannoma (*n* = 14, 3.6%), or primary CNS lymphoma (*n* = 13, 3.5%). The average tumor diameter was 36 mm, with most tumors located intraparenchymally, particularly in the temporal lobe (*n* = 48, 12.9%), frontal lobe (*n* = 36, 9.7%), or were multifocal (*n* = 41, 11%). The cerebellum (*n* = 18, 4.8%) and occipital lobe (*n* = 14, 3.8%) were least affected. Among extraparenchymal tumors, a location over the convexity was most common (*n* = 39, 10.5%), while the anterior cranial fossa was the least common location (*n* = 15, 4%).

### Univariable analysis

Among TEFs, male sex, underweight or normal BMI, increasing pack-years, and abnormal Na^+^/K^+^ levels were associated with an increased seizure incidence, whereas different ages and alcohol abuse demonstrated little to no isolated effects. Males had a higher seizure incidence of 35.8% (CI 35.1–36.5) than females (23.7%, CI 23.1–24.3), resulting in a OR of 1.79 (CI 1.14–2.82, *p* = 0.0113) for males. Among weight categories, obese patients showed the lowest seizure incidence (14%, CI 13–15) compared to overweight (25.4%, CI 24.6–26.2), normal weight (35.2%, CI 34.5–35.9), and underweight (42.9%, CI 40.7–45). Using obesity as the reference, normal weight and underweight patients had ORs of 3.34 (CI 1.5–8.52, *p* = 0.0058) and 4.61 (CI 1.44–15.54, *p* = 0.011). Smoking was linked to an increased risk (OR 1.015 per pack-year, CI 1.005–1.026, *p* = 0.003). Patients with pathological Na^+^/K^+^ levels had a seizure incidence of 39.2% (CI 38.1–40.3) versus 27.1% (CI 26.6–27.6) in patients with normal Na^+^/K^+^ levels (OR 1.73, CI 1.01–2.94, *p* = 0.0424).

Regarding, TIFs, while differences in seizure incidence were minor across histopathological subgroups, anatomical location revealed pronounced associations with TRE in the univariable analysis of TIFs. WHO grade 3/4 gliomas served as the reference histopathological category with a seizure incidence of 32.7% (CI 31.7–33.6). WHO grade 1/2 neuroepithelial tumors counted the highest incidence at 43.6% (CI 41.3–46.2), while only pituitary adenomas/craniopharyngiomas showed a significantly lower incidence of 6.3% (CI 5.4–7.1) with an OR of 0.14 (CI 0.02–0.5, *p* = 0.0092) compared to WHO grade 3/4 gliomas. Tumor diameter showed no association with TRE (OR 1.00, CI 0.987–1.014, *p* = 0.9425). For location, compared to the “other” reference group (14% incidence, CI 4.4–23.6), multifocal intraparenchymal tumors and those in the frontal, parietal, and temporal lobes had increased seizure incidences of 35–42% with ORs of 3.47–4.35. The central lobe had the highest incidence at 91.3% (CI 79.8–100) and an OR of 64.5 (CI 14.88–466.3, *p* < 0.0001). The low incidence (3.6%–9,5%) of extraparenchymal tumors in the cranial base were similar to the low seizure incidence (14%) of the reference group.

### Multivariable analysis

**Fig. 1:**
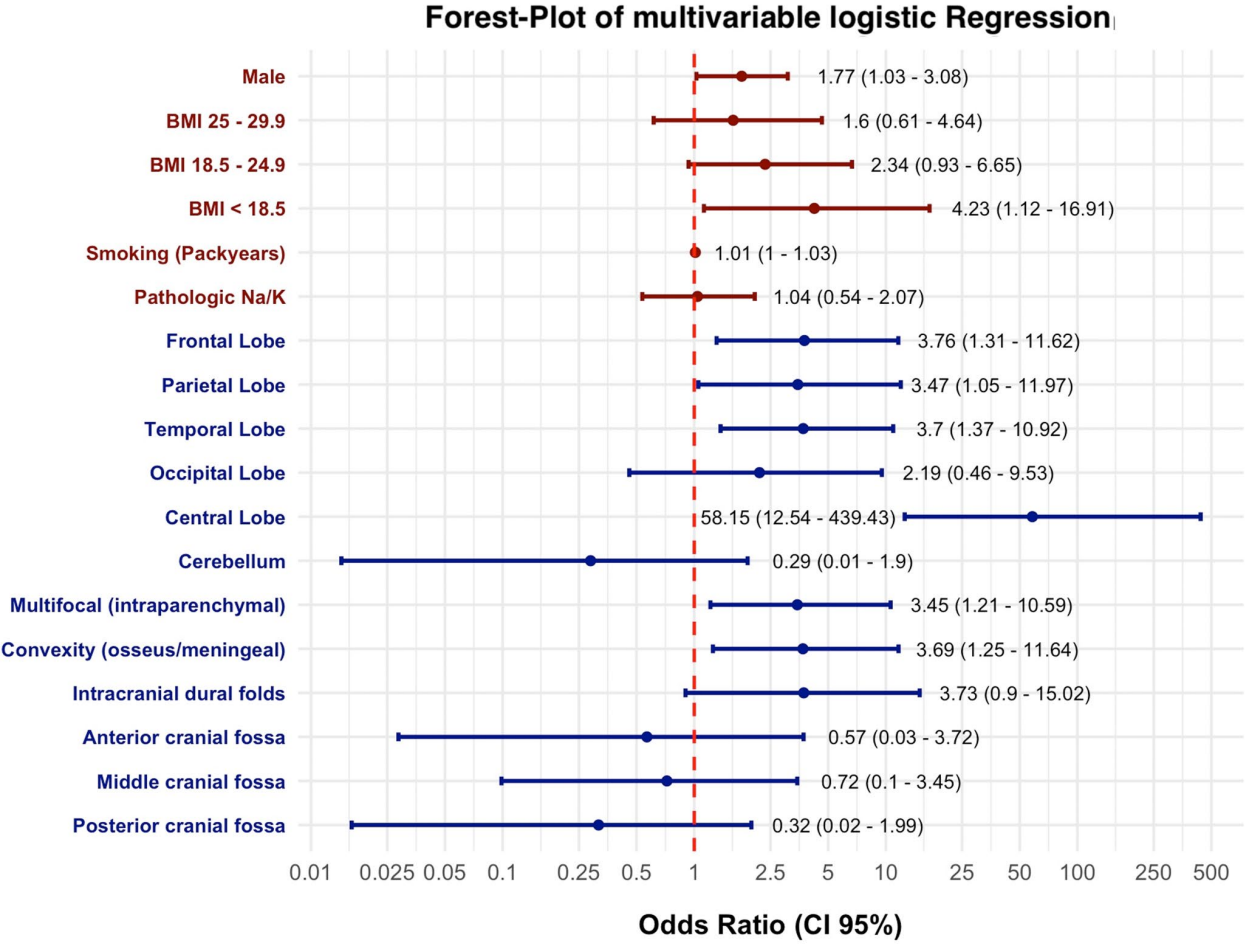
Forest plot displaying multivariable analysis (OR, 95% CI) of TIFs and TEFs, which included significant subcategories in univariable binary logistic regression. Reference subcategories (OR = 1) were: female (sex), BMI ≥ 30, normal Na^+^/K^+^ blood levels (= 135–145 mmol/L and 3.5–5.0 mmol/L), and “other” (anatomical location)

In contrast to the univariable model, the combined testing in the multivariable analysis (Fig. 1) revealed strong associations with TRE for the subgroups male sex (ref. female), underweight (ref. obese), increasing pack-years, most cortical regions, especially the central lobe, and multifocality (ref. “Other”) compared to their reference categories. Notably, normal BMI and abnormal Na^+^/K^+^ levels showed no, while extraparenchymal location in the osseous/meningeal convexity demonstrated secondarily significant associations, which could indicate existing multicollinearity or confounding among the tested independent variables.

**Fig. 2:**
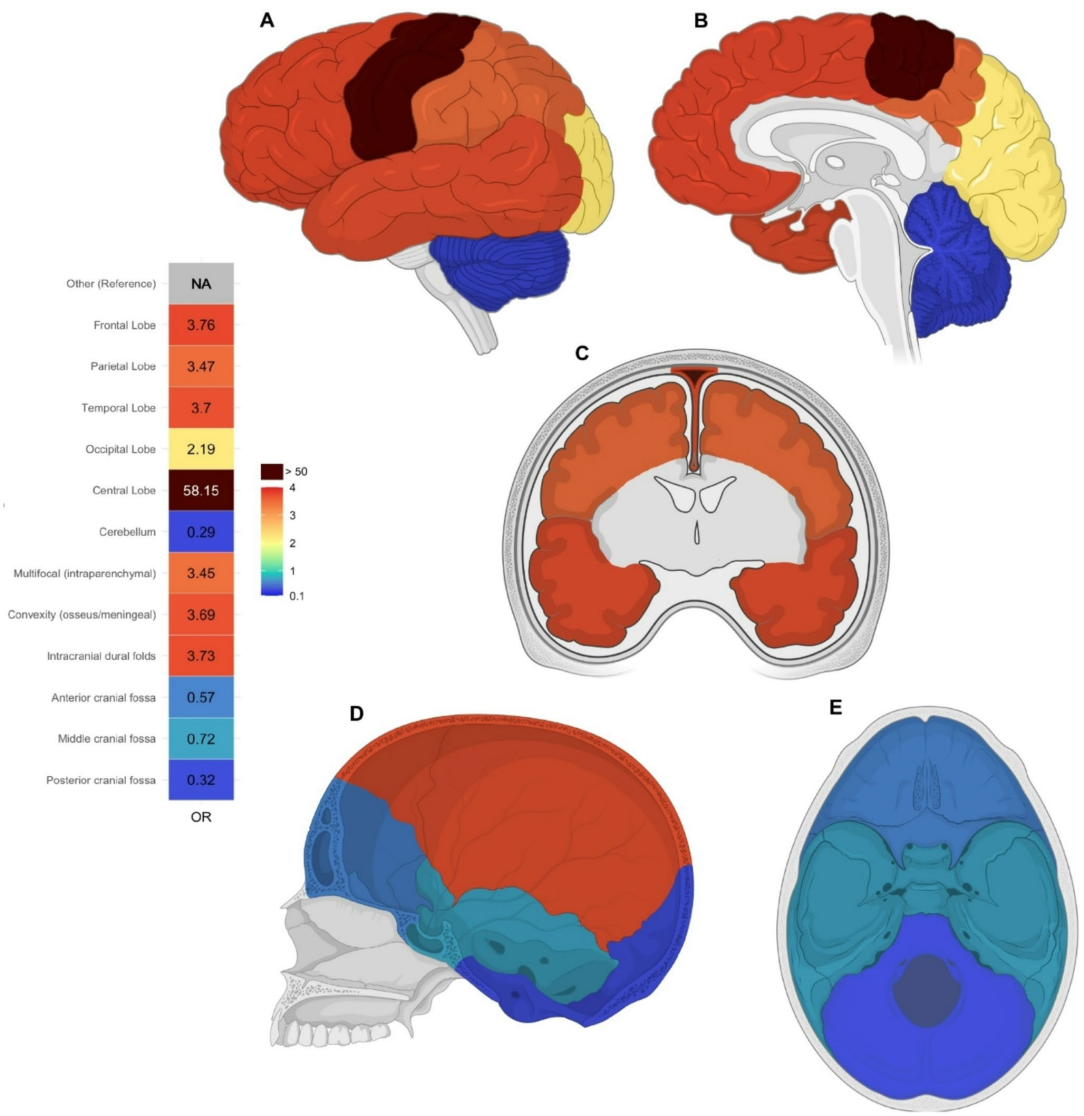
Heatmap visualizing the topography-specific ORs for TRE based on a multivariable regression model. Intraparenchymal and extraparenchymal brain tumor locations are compared to the reference “other” (shown as grey intraparenchymal regions in **A**,** B** and **C**)

Given the absence of relevant differences in seizure incidence and nonsignificant ORs within the subcategories of histopathology and tumor diameter in the univariable analysis, the multivariable analysis exclusively included anatomical location as TIF, which demonstrated effects comparable to those in the univariable model. Multifocal intraparenchymal tumors and location in the frontal, parietal, temporal, and central lobes, had significantly higher ORs than the reference category “other”. Extraparenchymal tumors in the osseous/meningeal convexity also showed a significant OR of 3.69 (CI 1.25–11.64, *p* = 0.0204). These findings are visualized in a topographic heatmap (Fig. 2)*.*

**A/B** lateral and medial views of brain hemisphere showing the frontal, parietal, temporal, occipital, central lobes and cerebellum.

**C** coronal cut at parietal lobe level (front-back axis) illustrating parietal, temporal lobes and falx cerebri (intracranial dural folds).

**D/E** sagittal and axial view of the skull and base presenting the osseus/meningeal convexity and different fossa allocations.

**Fig. 3:**
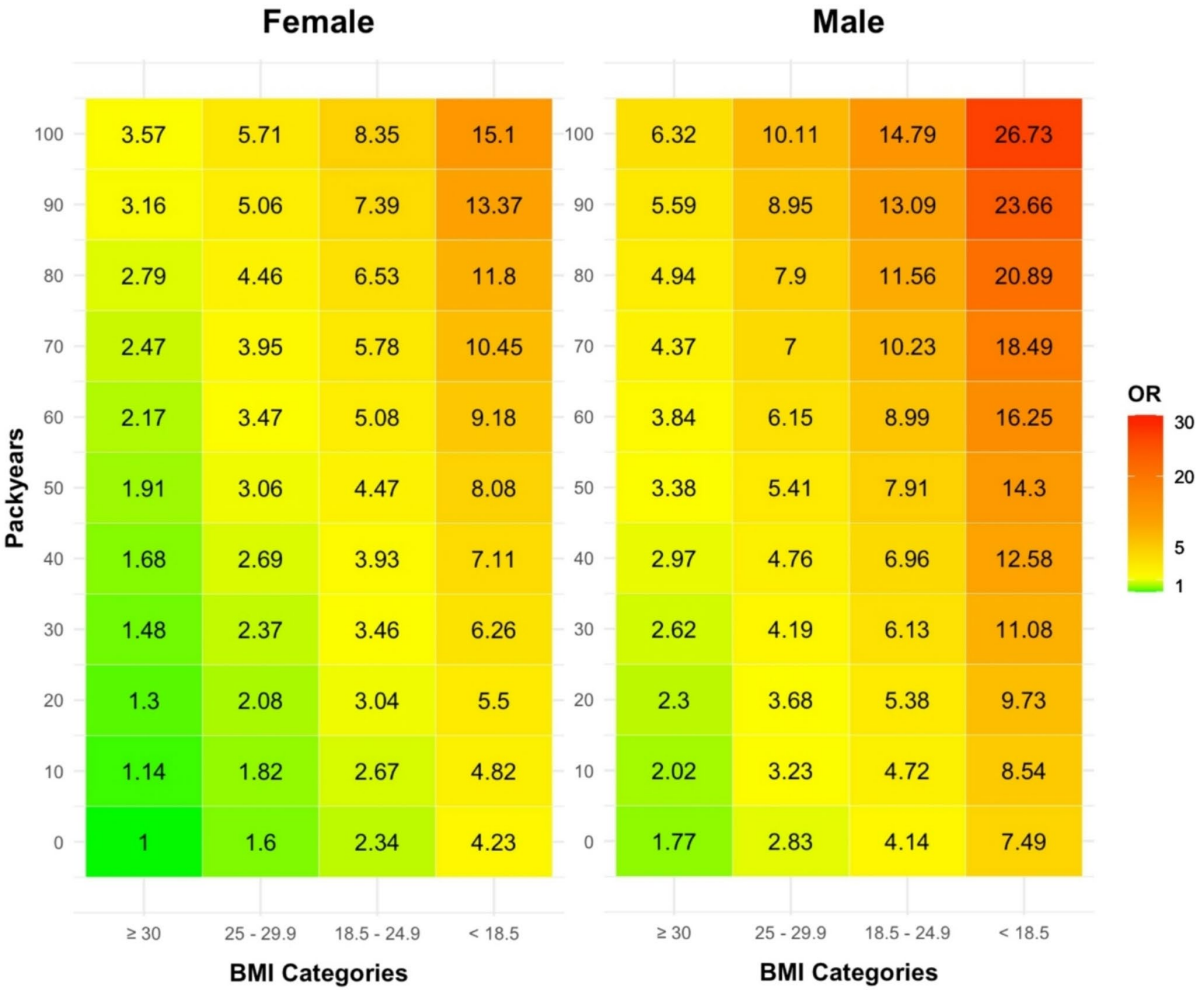
A TEF risk assessment heatmap calculates combined ORs of relevant TEFs from the multivariable analysis. A female, obese, non-smoking patient (OR 1) serves as the reference. In comparison, a male, underweight, heavily smoking patient (100 PY) has a calculated OR of 26.73. Depending on TEFs present in a patient, a combined OR can be described for the association with TRE

For TEFs, multivariable analysis revealed differences from the univariable results, notably the decrease in effect size and significance in the normal weight group compared to the obese reference (OR 3.34, *p* = 0.0058 vs. OR 2.34, *p* = 0.0851). The same effect was observed for pathological Na^+^/K^+^ (OR 1.73, *p* = 0.0424 vs. OR 1.04, *p* = 0.9098). In contrast, male sex (OR 1.77, CI 1.03–3.08, *p* = 0.0411) and smoking per pack-year (OR 1.013, CI 1.0–1.026, *p* = 0.0435) preserved their effect size and significance. A heatmap risk assessment tool combining relevant TEF ORs was generated (Fig. 3).

## Discussion

Our study underlines the previously reported strong association of specific TIFs with TRE, while additionally demonstrating the hitherto underexplored relevance of certain TEFs. Regarding histopathology, our data confirm that WHO grade 1/2 neuroepithelial tumors show higher, while pituitary adenomas/craniopharyngiomas and schwannomas exhibiting low seizure incidences, although smaller incidence differences and thus lesser significance among histopathological subgroups were noted compared to current literature [8, 11, 12, 17]. Anatomical location, especially the central lobe with its near-unconditional seizure association, plays a critical role in TRE risk, with the frontal, parietal, and temporal lobes also notably being rated as related lobar regions [1, 7–10]. Male sex, low BMI, and high smoking pack-years were identified as relevant independent TEFs associated with TRE.

Thus, our data support previous reports that seizure incidence vary widely across different tumor types [1, 8, 14, 17, 18, 24–27]. The overall seizure incidence in our cohort was 29.5%, aligning with the range of 30–50% [1]. WHO grade 3/4 gliomas showed incidences of 32.7%, consistent with the 20–40% reported in literature [17, 24, 25]. WHO grade 1/2 gliomas, typically associated with a 60–80% seizure risk [1, 8, 17, 18], exhibited a lower incidence (43.6%) in our study, with a non-significant OR of 1.61 compared to the reference group. This discrepancy may be attributed to the inclusion of low-risk (20%) ependymomas [17] in the group of WHO grade 1/2 neuroepithelial tumors. Primary CNS lymphomas, comprising 2–4% of CNS tumors, present seizures in about one-third of cases [26]. Our results showed a comparable subgroup occurrence (3.5%) and seizure incidence (38.5%). As with other tumors, cortical involvement significantly increases seizure risk [14, 26, 27]. Meningioma’s seizure incidence of 23.5% align with current data [17]. Their extraparenchymal location triggers seizres probably mainly via pressure effects and edema [27, 28], making them more epileptogenic at the convexity, where they interact with the cortex, than near the skull base. Metastases showed a seizure incidence of 40%, exceeding the reports of 10% or even lifetime risk of 35% [17, 29], potentially due to wide range of possible differences in histopathological origin, location or growth progression. Schwannoma and pituitary adenomas/craniopharyngiomas demonstrated low seizure incidences. Their peripheral locations limit cortical impact, aligning with available, but limited data [30], which again supports the hypothesis that anatomical location is more decisive than histopathological entity. Admittedly, since certain tumors occur preferentially in specific regions, it is almost impossible whether pathology or location drive differential risk.

We found no evidence for a correlation between tumor diameter and TRE. Instead, we confirmed that specific anatomical features, particularly a location in the central lobe, cortical involvement or intraparenchymal multifocality, exhibit a strong association with TRE. While some studies suggested an inverse association of tumor size and TRE [19, 20], others emphasized the importance of progression and growth over an isolated snapshot of tumor size [18]. Our data exceeds the previously reported strong association between anatomical tumor location and TRE incidence [2, 8–10, 12, 31]. Frontal, parietal, and temporal lobes — excluding the occipital lobe — showed similar seizure incidences (35–38%) with significantly higher ORs (3.4–3.8) in multivariable analysis compared to deeper subcortical structures. Lobar cortices, with their dense, highly interconnected neuronal networks, are prone to hyperexcitability and facilitated propagation, leading to unregulated discharges and thus TRE [32, 33]. Additionally, seizures in cortical regions, which are involved in conscious perception, may be more noticeable than those in subcortical areas that regulate automated processes. Subcortical regions also help suppress the spread of seizures in the cortex due to their unique connectivity patterns and lower synchronization, providing a natural inhibitory effect [34]. The central lobe warrants special attention: it exhibits an exceptionally high seizure incidence (91.3%) and OR (58.15) in multivariable analysis, surpassing all other factor in their association with TRE. This supports prior studies identifying the central lobe as a distinct seizure-related entity [11, 12, 15]. It remains unclear, however, whether the central lobe’s parenchyma is inherently more epileptogenic or whether seizures originating here may be recognized earlier due to more pronounced motor manifestations [15, 16, 35, 36]. These results must also be viewed in relative terms, as despite the clear statistical framework designed to increase statistical quality, the number of cases is generally considered as rather low. Furthermore, with regard to the high OR, the comparison group “Other” is a collective of various intraparenchymal and, above all, subcortical localizations, which are probably characterized by a lower incidence of TRE and thus inflate the OR of the central lobe in relative terms.

Intraparenchymal multifocality also emerged as a pro-epileptogenic feature, showing a seizure incidence of 41.5% and an OR of 3.45 in multivariable analysis, supporting a correlation between tumor burden and TRE [7]. Upper extraparenchymal tumors, such as meningiomas, presented seizure incidence around 30% with an OR about 3.7, likely due to their interaction with the cerebral cortex, particularly when arising from dural folds or located in the convexity regions. In contrast, cranial base meningiomas exhibited lower seizure incidences and ORs, likely due to less direct cortical impact and their positioning over less epileptogenic brain areas.

In addition to TIFs, TEFs, particularly male sex, low BMI, and smoking were associated with TRE. Based on the effect size of the ORs for alcohol abuse and the additional evidence level for pathological Na^+^/K^+^ levels in the univariable analysis, our study suggests a possibly interrelated association of these variables with TRE. Aside from basic characteristics like age and sex, patient-specific factors related to TRE have been underexplored. Some studies suggested associations of male sex and younger age with seizure risk [18–21, 26, 29]. Our results confirm male sex as an independent risk factor, though no significant differences were observed among ages. For BMI, our results indicate a protective effect for overweight and obese individuals compared to lower BMI. Underweight patients, with a seizure incidence of 42.9%, appear at significantly higher risk. This aligns with studies linking extreme BMI, including underweight, to increased seizure risk in TRE [37]. Conversely, while obesity has been associated with drug-resistant epilepsy and cognitive decline in idiopathic epilepsy, its role in TRE remains less clear [38–40]. Smoking was associated with increased seizure incidence, with a notable OR rise per pack-year (OR 1.013) in multivariable analysis. Alcohol abuse also showed an elevated seizure incidence, but lacked statistical significance in our study, as in a similar Italian study [41]. Nevertheless, further investigation remains reasonable, given that irregular and heavy alcohol consumption, especially withdrawal, generally lower seizure thresholds [42, 43]. Existing data mostly link smoking and thus elevated CO-Hb levels [44–46], as well as excessive alcohol consumption, to increased seizure risk in primary epilepsy [46]. It is conceivable that smoking, low BMI and alcohol abuse may increase general seizure susceptibility rather than specifically triggering TRE. Furthermore, potential confounding must always be taken into account, for example through the association of smoking with tumor etiology. However, metastatic tumors were not associated with increased TRE risk in our cohort, while smoking remained significant, suggesting that this effect is unlikely to be explained solely by tumor type. Therefore, future studies should aim to disentangle TRE-specific mechanisms from confounding and seizure risk factors observed in the general population. Finally, we suspected an association between Na^+^/K^+^ alterations and TRE, since this may disrupt neuronal action potentials and affect excitability, and play a role in inhibitory GABA homeostasis via K^+^Cl^−^ (KCC2) and Na^+^K^+^2Cl^−^ (NKCC) co-transporters [7]. While pathological Na^+^/K^+^ levels were linked to increased seizure incidences in univariable analysis (OR 1.73), this was not confirmed in multivariable analysis.

Although the findings regarding TEFs and their association with TRE appear compelling, they should currently be regarded primarily as a conceptual stimulus for potential clinical application, as their validity and relevance must first be verified in future prospective studies. The prophylactic use of antiseizure medication is still discouraged due to an unsatisfactory risk–benefit and side-effect profile [2–5]. In terms of lifestyle modification, however, a pragmatic and effective approach for patients with a high-risk TEF profile may be to abstain from activities that pose potential harm to themselves or others, such as driving or operating heavy machinery, and to ensure close clinical follow-up.

This study has its limitations, particularly due to the limited cohort and sample size for subgroups, which limits the number of independent variables included and necessitates the exclusion of other interesting and relevant TIFs and TEFs such as molecular markers, peritumoral edema, and other blood values e.g. calcium and magnesium. Furthermore, peritumoral edema was not assessed due to methodological heterogeneity, and should be considered in prospective studies using standardized imaging protocols. Yet, the clinical data were systematically collected in a standardized, prospective workflow, ensuring high completeness and minimal missing information. Observer-dependency in image analysis was addressed by having two blinded raters and a strictly defined binary topographic-anatomical classification. Given the exploratory nature of the study and scarce literature on TEFs, the proposed association remains preliminary, requiring further validation and refinement.

## Conclusion

This study aimed to explore the interplay of specific TIFs and TEFs with TRE. Among the TIFs, a cortical location, especially in the central lobe, emerged as the most significant and independent association with TRE. Among the TEFs, male sex, low BMI and smoking with increasing pack-years revealed also strong associations with TRE. Our findings underline the importance of developing a holistic risk assessment including TIFs and TEFs to guide clinical decision and patient lifestyle modification.

## Supplementary information

Below is the link to the electronic supplementary material.

Supplementary file 1 (docx 11.7 KB)

## Data Availability

All data and code generated or analyzed during this study are included in this published article and its supplementary information files.

## Code Availability

All data and code generated or analyzed during this study are included in this published article and its supplementary information files.
