# Supplementary material for: Intrinsic and extrinsic risk factors in tumor-related epilepsy
Source: Neurosurg Rev. 2025 Dec 26;49(1):88. doi: 10.1007/s10143-025-04020-z (PMC12743018; doi:10.1007/s10143-025-04020-z)
Supplement: Supplementary file 1 — (docx 11.7 KB) [file 10143_2025_4020_MOESM1_ESM.docx]

| **Supplemental methods**  MRI images generally fulfilled the following minimal requirements: 3 Tesla Skyra VD13 (Siemens, Erlangen, Germany) using a 32-channel head coil with the following parameters: a high-resolution 3D T1-weighted anatomical sequence planned on the ACPC line plus 20° on a sagittal image with voxel size: 0.8 × 0.8 ×1.0 mm^3^ with a Field of View 230×230 mm^2^ and resolution of 288 × 288. 176 slices per slab with a thickness of 1 mm, TR/TE 2200/5.14 ms, TI 900 ms, flip angle 8°. FLAIR images acquired with the same orientation as the T1-weighted images. The acquisition parameters were as followed 0.9 × 0.9 × 1.0 mm^3^ with a Field of View 230 × 230 mm^2^ and resolution of 256 × 256, 176 slices per slab with a thickness of 1 mm, TR/TE 4000/387 ms, TI 1800 ms.  **Supplemental results** | | | | | |
| --- | --- | --- | --- | --- | --- |
| **Supplemental Table 1** Odds ratios from multivariable analysis. Tested tumor-extrinsic and -intrinsic risk factors containing factors with mainly significant subcategories (p value < 0.05) in univariable analysis. | | | | | |
| Risk factor |  |  |  | Odds ratio (CI 95%) | P value |
| *Tumor-extrinsic* |  |  |  |  |  |
| Sex  Female (ref.)  Male |  |  |  | 1 (ref.)  1.77 (1.03 - 3.08) | **0.0411** |
| BMI groups  ≥ 30: Obese (ref.)  25 - 29.9: Overweight  18.5 - 24.9: Normal  < 18.5: Underweight |  |  |  | 1 (ref.)  1.6 (0.61 - 4.64)  2.34 (0.93 - 6.65)  4.23 (1.12 - 16.91) | 0.3586  0.0851  **0.0353** |
| Smoking pack-years (cont.) |  |  |  | 1.013 (1 - 1.026) | **0.0435** |
| Pathological Na^+^/K^+^ blood value  No (ref.)  Yes |  |  |  | 1 (ref.)  1.04 (0.54 - 2.07) | 0.9098 |
| *Tumor-intrinsic* |  |  |  |  |  |
| Location  Other (ref.)  Frontal lobe  Parietal lobe  Temporal lobe  Occipital lobe  Central lobe  Cerebellum  Multifocal (intraparenchymal)  Convexity (osseus/meningeal)  Intracranial dural folds  Anterior cranial fossa  Middle cranial fossa  Posterior cranial fossa |  |  |  | 1 (ref.)  3.76 (1.31 - 11.62)  3.47 (1.05 - 11.97)  3.7 (1.37 - 10.92)  2.19 (0.46 - 9.58)  58.15 (12.54 - 439.43)  0.29 (0.01 - 1.9)  3.45 (1.21 - 10.59)  3.69 (1.25 - 11.64)  3.73 (0.9 - 15.02)  0.57 (0.03 - 3.72)  0.72 (0.1 - 3.45)  0.32 (0.02 - 1.99) | **0.0163**  **0.0429**  **0.0124**  0.3019  **< 0.0001**  0.2711  **0.0236**  **0.0204**  0.0626  0.6132  0.7041  0.2997 |
